# The Evolution of Manufacturing Technology for GaN Electronic Devices

**DOI:** 10.3390/mi12070737

**Published:** 2021-06-23

**Authors:** An-Chen Liu, Po-Tsung Tu, Catherine Langpoklakpam, Yu-Wen Huang, Ya-Ting Chang, An-Jye Tzou, Lung-Hsing Hsu, Chun-Hsiung Lin, Hao-Chung Kuo, Edward Yi Chang

**Affiliations:** 1 Department of Photonics, Institute of Electro-Optical Engineering, National Yang Ming Chiao Tung University, Hsinchu 30010, Taiwan; arsen.liou@gmail.com (A.-C.L.); itriA30378@itri.org.tw (P.-T.T.); cath01.ee09@nycu.edu.tw (C.L.); huangwendy227@gmail.com (Y.-W.H.); s922085493@gmail.com (Y.-T.C.); 2 Industrial Technology Research Institute, No. 195, Sec. 4, Chung Hsing Rd., Chutung, Hsinchu 31040, Taiwan; 3 Taiwan Semiconductor Research Institute, No. 26, Prosperity Road 1, Hsinchu 30078, Taiwan; jerrytzou.ep00g@gmail.com; 4 International College of Semiconductor Technology, National Yang Ming Chiao Tung University, Hsinchu 30010, Taiwan; edc@mail.nctu.edu.tw; 5 Semiconductor Research Center, Hon Hai Research Institute, Taipei 114699, Taiwan

**Keywords:** gallium nitride, high-electron mobility transistor, CMOS-compatible Au-free process

## Abstract

GaN has been widely used to develop devices for high-power and high-frequency applications owing to its higher breakdown voltage and high electron saturation velocity. The GaN HEMT radio frequency (RF) power amplifier is the first commercialized product which is fabricated using the conventional Au-based III–V device manufacturing process. In recent years, owing to the increased applications in power electronics, and expanded applications in RF and millimeter-wave (mmW) power amplifiers for 5G mobile communications, the development of high-volume production techniques derived from CMOS technology for GaN electronic devices has become highly demanded. In this article, we will review the history and principles of each unit process for conventional HEMT technology with Au-based metallization schemes, including epitaxy, ohmic contact, and Schottky metal gate technology. The evolution and status of CMOS-compatible Au-less process technology will then be described and discussed. In particular, novel process techniques such as regrown ohmic layers and metal–insulator–semiconductor (MIS) gates are illustrated. New enhancement-mode device technology based on the p-GaN gate is also reviewed. The vertical GaN device is a new direction of development for devices used in high-power applications, and we will also highlight the key features of such kind of device technology.

## 1. Introduction

**Figure 1:**
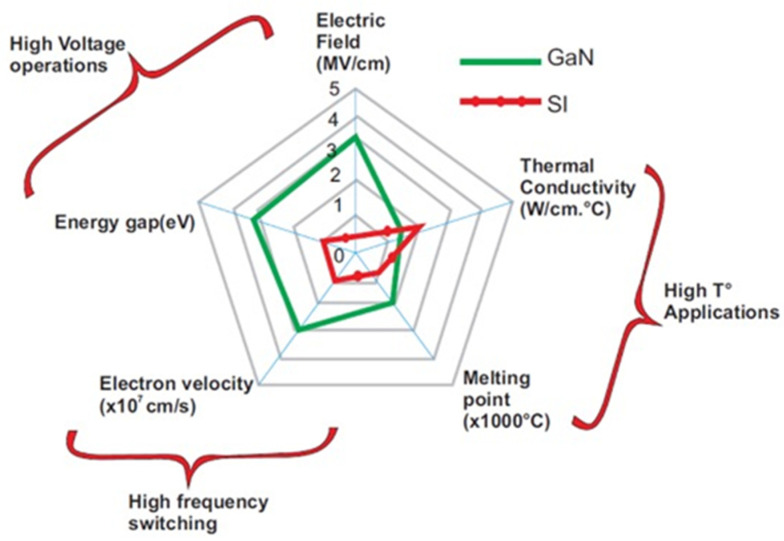
Differences in material properties between GaN and Si [2]. (Data from [2]).

Gallium nitride (GaN) is a high-potential semiconductor material. It has been used to fabricate high-electron mobility transistors (HEMTs) for applications in power devices and radio frequency (RF) power amplifiers because of its superior material characteristics compared with silicon (Si)-based materials, including a wide bandgap, high breakdown electric field, and high electron saturation velocity, as shown in Figure 1 [1,2].

In 1979, Takashi Mimura invented the gallium arsenide high-electron mobility transistor (GaAs HEMT) [3]. An HEMT uses a heterojunction to enhance electron mobility, thereby increasing the speed of electron transport. A heterojunction with a wider-bandgap Schottky barrier and a lower-bandgap channel can be used to form a modulation-doping structure to spatially separate conducting electrons from their doped impurity atoms. Therefore, a transistor with a high-electron mobility channel can be created (i.e., HEMT). The GaAs HEMT has been widely used in mobile phones, satellite TV receivers, and radar equipment [4,5,6]. The aluminum gallium nitride (AlGaN)/gallium nitride (GaN) heterojunction was first reported in 1991 [7], and the first AlGaN/GaN HEMT was subsequently developed in 1993 [8,9], where superior channel electron mobility was demonstrated. In 2006, the GaN HEMT RF transistor was first produced by Eudyna of Japan [10]. Since then, other companies have also announced various GaN HEMT products for RF applications. The GaN HEMT can be operated at 50 V with an output power over 200 W for mobile communication applications using silicon carbide (SiC) as the substrate [11,12,13]. In 2001, a research team from the University of California at Santa Barbara reported a GaN HEMT for power switch application fabricated on SiC, which possessed a breakdown voltage higher than 1000 V [14]. Since then, GaN HEMTs have been studied intensively on Si substrates for their low cost, high volume, and high-performance power switch applications. After years of development, the state-of-the-art GaN HEMT power devices on Si can be operated at a breakdown voltage higher than 1200 V [15,16].

**Figure 2:**
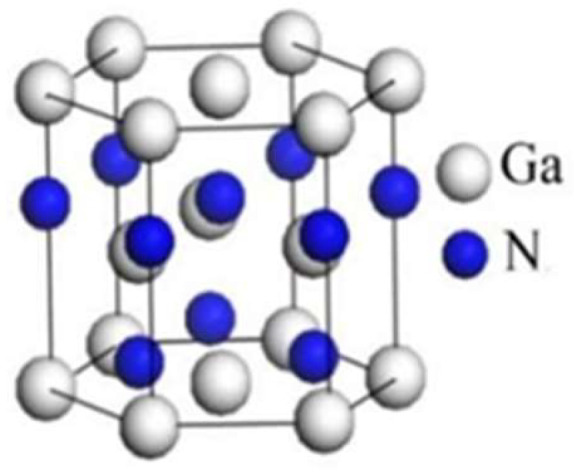
Gallium nitride wurtzite structure [18]. (Data from [18]).

Unlike the GaAs HEMT counterpart which needs to have a doping layer in the wide-bandgap AlGaAs barrier, GaN HEMTs do not need a doping layer in the wide-bandgap AlGaN barrier layer. Due to the polarization effects of the hexagonal wurtzite structure of AlGaN and GaN materials, the heterostructure of AlGaN/GaN has a high-density two-dimensional electron gas (2D electron gas; 2DEG) formed at the interface between AlGaN and GaN [17]. These polarization effects include two mechanisms, one is spontaneous polarization (P_SP_) and the other is piezoelectric polarization (P_PE_). Spontaneous polarization (P_SP_) is induced because gallium atoms in the non-centrosymmetric wurtzite structure do not locate at the center of the mass with respect to nitrogen atoms. On the other hand, the piezoelectric polarization effect (P_PE_) is induced because of the stack of two lattice-mismatched wurtzite III-nitride materials. Polarization charges are formed due to the mismatch strain at the heterogeneous junction, as shown in Figure 2 [18].

**Figure 3:**
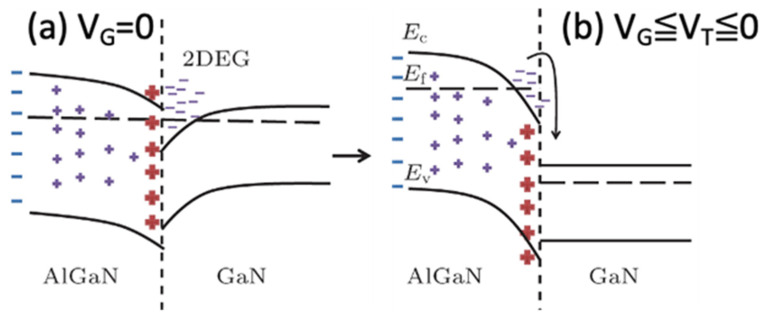
(**a**) The charge accumulation at the potential well as V_G_ = 0, and (**b**) the accumulated charges depleted as V_G_ < V_T_ < 0 [19]. Figure reproduced with permission from Chin. Phys. B.

The most commonly used GaN HEMT is an AlGaN/GaN heterostructure. The typical thickness of AlGaN is around 20–25 nm, and the thickness of the GaN channel and buffer layer is around 1–5 µm depending on the requirement of the breakdown voltage [15]. According to the energy band structure of the GaN HEMT, there is a potential energy well at the junction of AlGaN and GaN, and there will be a transportable energy state formed by the accumulation of electrons in this energy well, as shown in Figure 3 [17,19]. Figure 3a shows the charge accumulated in the potential well as V_G_ = 0, and Figure 3b indicates that the accumulated charges are depleted with V_G_ < V_T_ < 0.

**Figure 4:**
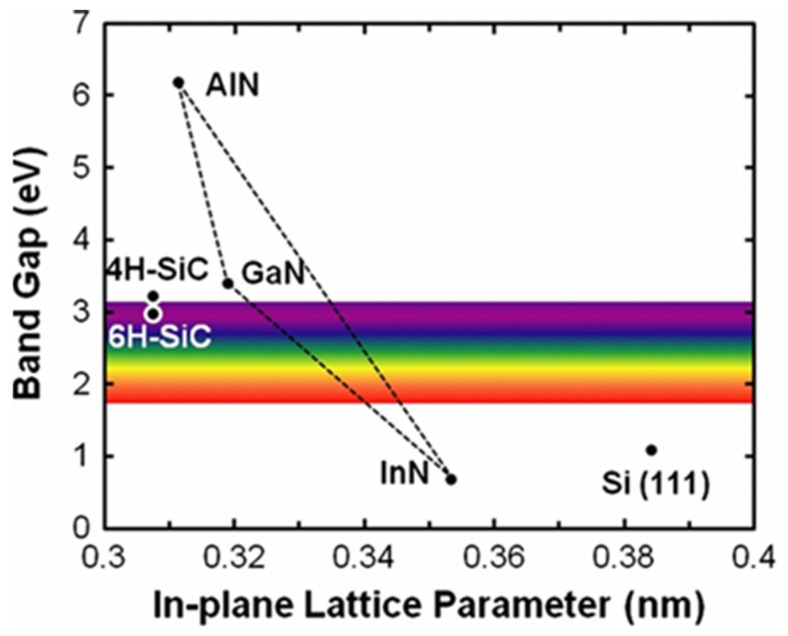
Lattice parameters and band gap for GaN-AlN-InN alloys [22]. (Data from [22]).

In addition to the AlGaN/GaN HEMT, the InAlN/GaN heterostructure also attracts a lot of attention [20,21]. For AlGaN grown on GaN, the lattice mismatch restricts the AlGaN content and thickness. Figure 4 demonstrates the bandgaps versus lattice parameters of AlN-GaN-InN compounds [22]. InAlN, with around 18% In, is lattice-matched with GaN, and its wide bandgap makes it an ideal barrier. In the meantime, the heterostructure of InAlN/GaN possesses high accumulation charges at the interface owing to the larger difference in spontaneous polarization between the two layers. In Section 2.1, we will further discuss the current development of the GaN epitaxial structure.

### 1.1. Radio Frequency HEMT Device Applications

**Figure 5:**
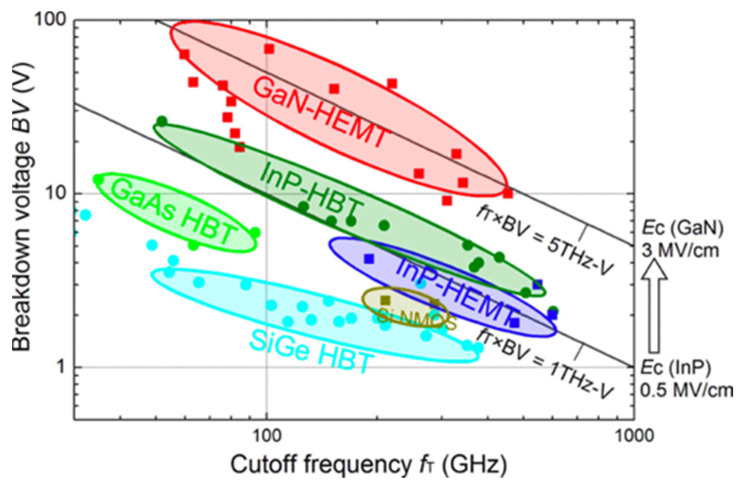
Comparison of breakdown voltage and cut-off frequency among various high-speed device technologies [32]. Figure reproduced with permission from IEEE Trans. Electron Devices.

In the past two decades, mobile communication technology has developed rapidly from 2G in 1990 to the introduction of 3G in 2000, and then the deployment of 4G service in 2011 until today [23,24,25,26,27,28,29]. Under the trend of continuous innovation and service demand, 5G was launched recently in 2019–2020. Global research institutions have also begun to invest in research and development for 5G +/ 6G technologies. Currently, 5G is still operated below 6 GHz which is relatively similar to 4G. High-band 5G operating at mmW (28–40 GHz) is still in development and is expected to be deployed in the near future [23,27,30]. The GaN HEMT that can be applied in high-frequency power amplifiers has been regarded as an important device technology by various manufacturers. Figure 5 shows the breakdown voltage versus current gain cut-off frequency (f_T_) of different types of devices [31,32]. The GaN HEMT shows the best capabilities, combining a high voltage, high power, and high speed. Moreover, the GaN-on-Si technology that can greatly reduce production costs and is more suitable for high-volume production has attracted a lot of attention [33]. In the past, the GaN HEMT was mainly fabricated on SiC substrates for high-frequency applications owing to the better quality of epitaxy and the better heat dissipation. With improved techniques in epitaxial growth and layout optimization for GaN-on-Si devices, several manufactures have announced the launch of the mass production of GaN-on-Si technology for high-frequency applications [34].

In view of the applications in 4G to 5G base stations, outdoor WiFi, millimeter-wave (mmW) small cells, and other high-data rate wireless communication applications, increasing the transmit power and efficiency is an important issue. At present, RF and mmW power amplifiers in the market mainly use SiGe- or GaAs-based transistors. However, due to the constraint in the breakdown voltage, individual power amplifiers made of these materials cannot provide an efficient output power in the mmW band [31]. Technologies such as phased array antennas are needed to achieve the overall required transmission power [35]. However, too many phase array antennas will cause problems such as narrow beams and manufacturing complexity. Therefore, further improvement in the output power and efficiency of the devices for power amplifiers has become an important issue.

In recent years, a GaN HEMT with a maximum oscillating frequency (f_max_)/current gain cut-off frequency (f_t_) of up to 300 GHz has been demonstrated [36]. An MMIC fabricated using GaN technology with an operating frequency up to the G band was also presented. The output power and gain can reach around 16 dBm and 12 dB at 181 GHz with 5.5% of power-added efficiency (PAE) [37]. For 5G operation, an output power density of 10 W/mm at sub-6 GHz and an output power density of 6 W/mm at 40 GHz were achieved [38]. Moreover, Intel has demonstrated a GaN MOSHEMT with f_t_/f_max_ reaching 200/350 GHz and a high mmW (28 GHz) output power of 19.5 dBm fabricated on Si. Three-dimensional integration with CMOS has also been reported [33,39].

### 1.2. Power HEMT Device Applications

**Figure 6:**
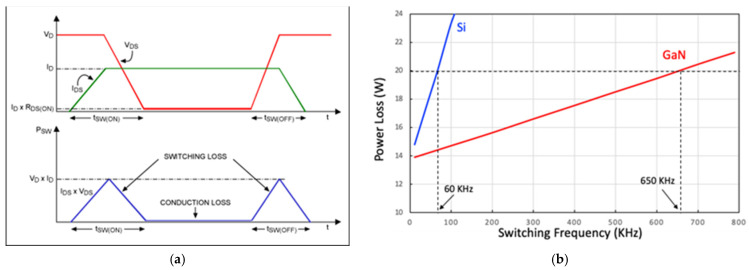
(**a**) Relation between switching speed and energy loss for a power switch, and (**b**) the lower power loss of GaN compared with Si [40]. Figure reproduced with permission from IEEE 2016 10th International Conference on Compatibility, Power Electronics and Power Engineering.

For power device applications, GaN HEMTs have shown low on-resistance to greatly reduce the conduction loss of the switch with a high breakdown voltage. Moreover, GaN power devices have lower parasitic capacitance, which can provide faster switching than silicon power transistors, meaning they have a much lower energy loss. The relation between the switching speed and energy loss of power switches is depicted in Figure 6a. The switching speed of GaN is faster; hence, the switching loss of the GaN HEMT is much less than that of Si, as indicated by Figure 6b [40]. Therefore, the GaN HEMT has great potential in high-speed, high-power switch applications.

Overall speaking, the GaN-on-Si HEMT is still inferior to SiC devices in terms of high-voltage and high-power performances owing to the higher defects in epitaxy and the worse thermal dissipation capability of Si substrates. However, GaN is expected to replace Si-based diodes, MOSFETs, and other power components in the low- and medium-power fields in the future. It is predicted that SiC has an advantage over GaN above 900 V; however, GaN is very competitive for operating voltages below 1000 V due to the benefits of a low switching loss and lower cost [41].

Currently, the fastest-growing GaN power device application is GaN fast chargers, and various products have been brought to the market. At present, fast chargers with power ranging from 65~125 W have gradually become mainstream products because the fast charger made by a GaN power IC is small in size, easy to carry, capable of high-power operation, has a higher energy efficiency, and is cost-effective.

### 1.3. Process Development (From Au-Based to Au-Free μS-Coμpatible)

Traditionally, the manufacturing of GaN HEMT devices is based on Au-based metallization schemes, including ohmic contact metals and Schottky metal gates. In the past decade, due to the rapid development of GaN epitaxy on silicon substrates, the development of complementary metal–oxide–semiconductor (CMOS)-compatible fabrication processes for GaN-on-Si devices has increased sharply and gradually matured. There are several major changes in fabrication techniques as compared to the CMOS-compatible processes with conventional III–V processes.

For instance, CMOS-compatible processes usually need to have a planarized structure. Therefore, ion implantation of nitrogen or other inert elements to amorphized non-active regions to achieve device isolation instead of etching the active layers (i.e., “mesa etching”) can be a preferred method. On the other hand, most traditional III–V devices use Au-based metallization schemes that are incompatible with the CMOS fabrication process. To be compatible with the CMOS fabrication process, the commonly used Ti/Al/Ni/Au ohmic contact metal stack can be changed to Ti/Al/Ni/TiN [42]. Moreover, a TiN diffusion barrier/Schottky metal and a Cu or Al conductor layer can be used for the metal gate instead of a Ni Schottky metal and Au conductor layer [43].

In this article, we will first describe the key steps in traditional Au-based manufacturing processes for GaN HEMT devices including the ohmic contact at the source/drain area, and the Schottky metal gate. Then, the evolution of the Au-free process flow for CMOS-compatible GaN technology will be illustrated. We will also discuss the new developments in structures and materials used in CMOS-compatible manufacturing processes in ohmic contact formation and gate structures [44,45].

## 2. Conventional GaN HEMT Technology

### 2.1. Epitaxy

In recent years, the epitaxial quality of GaN and its doping technology have become matured and resulted in the fast development of high-power and high-frequency electronic devices. Due to the lack of high-quality and large-size GaN substrates on the market, GaN heterostructures are mainly grown on silicon (Si), sapphire (Sapphire), or silicon carbide (SiC) substrates.

During the epitaxy process, the mismatch in the lattice and thermal expansion coefficient between GaN and the substrates is the key factor to be concerned about. Listed in Table 1 are the physical parameters of the commonly used substrates [46]. The lattice constant and thermal expansion coefficient mismatch between the SiC substrate and the GaN is the smallest; therefore, the quality of the GaN epitaxy grown on SiC is the best. Moreover, the SiC substrate has very good thermal conductivity, meaning it can effectively remove the heat generated by GaN components during high-frequency and high-power operations to enhance reliability. High-quality GaN-based materials coupled with a substrate with good thermal conductivity could improve the overall characteristics of the device. However, the SiC substrate is an expensive substrate that is difficult to produce; therefore, the cost-effective GaN-on-Si technology becomes an attractive choice for many manufacturers. However, due to the larger mismatch in the lattice constant and thermal expansion between Si substrates and GaN, it is more difficult to grow high-quality GaN on Si substrates. Usually, it is necessary to use a thicker or complex buffer layer structure [47,48,49].

**Figure 7:**
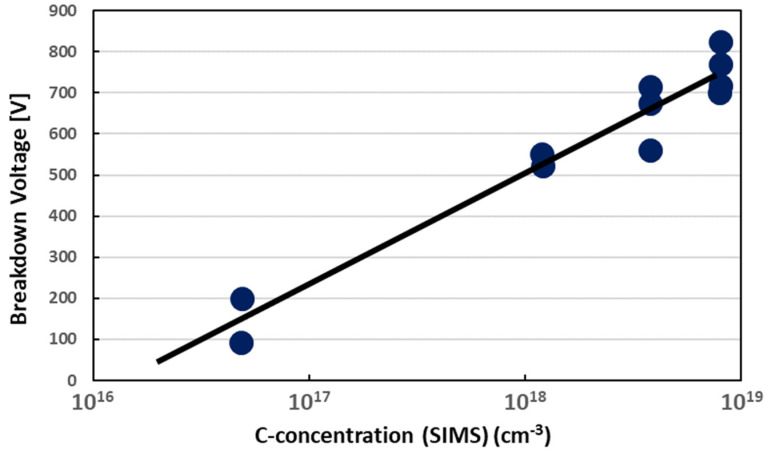
The relationship between breakdown voltage and Carbon doping concentrations in the GaN buffer layer [53]. Figure reproduced with permission from J. Cryst. Growth.

The design and growth of buffer layers are very critical. The characteristics of the GaN HEMT, especially the breakdown voltage, are affected by the quality and resistivity of the underlying buffer layer. The GaN buffer layer under the channel needs higher resistivity to prevent the DC leakage current and AC coupling. Since undoped GaN is typically n-type, adding a p-type dopant is required to obtain a highly resistive buffer. A commonly used p-type dopant is Mg; however, the memory effect of Mg is very strong [50], and it will affect the properties of the subsequent AlGaN/GaN epitaxy. Thus far, Fe and carbon are used as p-type dopants for the buffer layer. However, Fe dopants still have the issue of a memory effect on the MOCVD growth, and it is not easy to obtain an abrupt interface [51,52]. Carbon is a more attractive p-type dopant for the buffer layer. It does not have a strong memory effect, and its concentration and the breakdown voltage of the buffer layer can be adjusted by changing the epitaxial conditions, as shown in Figure 7 [53,54]. On the other hand, a buffer layer has to be designed to release stress and make the surface flat. Typical structures include a graded AlGaN buffer [55], a GaN/AlGaN superlattice buffer [56], low-T GaN, or low-T GaN with an AlN insertion [57]. For the growth of GaN on Si substrates, AlN nucleation and a buffer layer have to be grown to avoid interaction between Ga and Si at high growth temperatures.

**Figure 8:**
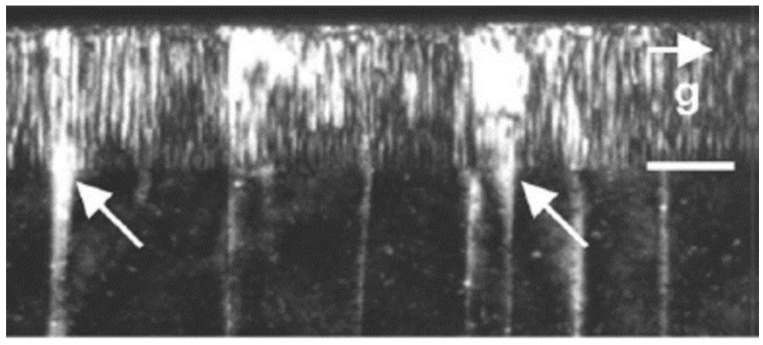
Cross-sectional TEM images of InAlN layer showing phase separation [58]. Figure reproduced with permission from Appl. Phys. Lett.

The typical top barrier layer for GaN HEMTs is AlGaN or InAlN, as described in Section 1. While the AlGaN barrier layer is very mature, the thickness and Al content have restrictions due to the lattice mismatch with GaN. As the thickness becomes too thin, the charge in the channel would decline due to insufficient piezoelectric polarization. On the contrary, defects would appear as the thickness exceeds the critical thickness. The lattice-matched InAlN (18% In) barrier has stronger spontaneous polarization to induce a much higher channel charge than AlGaN [20]. Nevertheless, phase separation of InN and AlN could occur during MOCVD growth and result in a high gate leakage current, as the TEM image shows in Figure 8 [58,59,60]. Careful optimization of the growth condition has to be carried out to avoid phase separation.

### 2.2. Ohmic Contact

An ohmic contact is a type of metal/semiconductor contact formed at the source/drain region. The interface has to be heavily doped to form a very thin energy barrier to allow for the tunneling of carriers through the interface to obtain low contact resistance. In the traditional III–V-based process, the most commonly used ohmic contact metals for GaN are diffusion-type multi-layer ohmic contact metals based on titanium (Ti)/aluminum (Al). Then, nickel (Ni)/gold (Au) is stacked on Ti/Al to form a thick conductive metal layer [61]. For a CMOS-compatible process, Ni/Au is not used, but TiN, Al, or Cu is used for the thick conductive metal layer. Generally, there is a diffusion barrier layer (i.e., Ni) between the thick conductive metal layer and the Ti/Al ohmic contact metal, meaning the top conductive layer does not affect the characteristics of the GaN ohmic contact interface.

**Figure 9:**
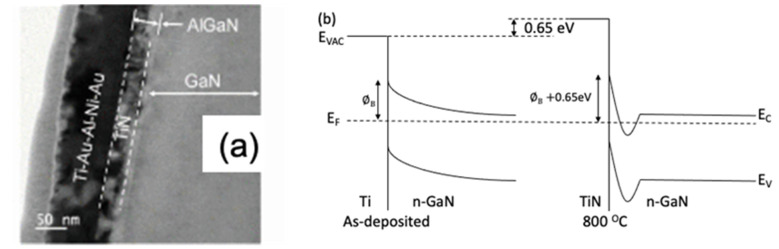
(**a**) TEM images of Ti/Au/Al/Ni/Au structure after alloying [63], and (**b**) band diagram before/after alloying [65]. Figure reproduced with permission from IEEE Electron Device Lett. & AIP Publishing.

The role of Ti/Al diffusion-type ohmic contact metals in the process of reducing the interface energy barrier is explained by several studies in the literature [61,62,63,64]. When rapid thermal annealing (RTA) is used, and the maximum temperature is raised to above 800 °C, Ti/Al can diffuse into the GaN layer and form a uniform TiAlN alloy. The process can cause nitrogen vacancies to be generated in the crystal lattice. Nitrogen vacancies in GaN act as the n-type dopants to enhance the n-type characteristics of GaN to further reduce the resistance. Figure 9a shows the TEM images formed by annealing a Ti/Au/Al/Ni/Au ohmic contact metal at 850 °C [63]. Figure 9b exhibits the effect of N vacancy formation on the interfacial band structure of GaN [65].

**Figure 10:**
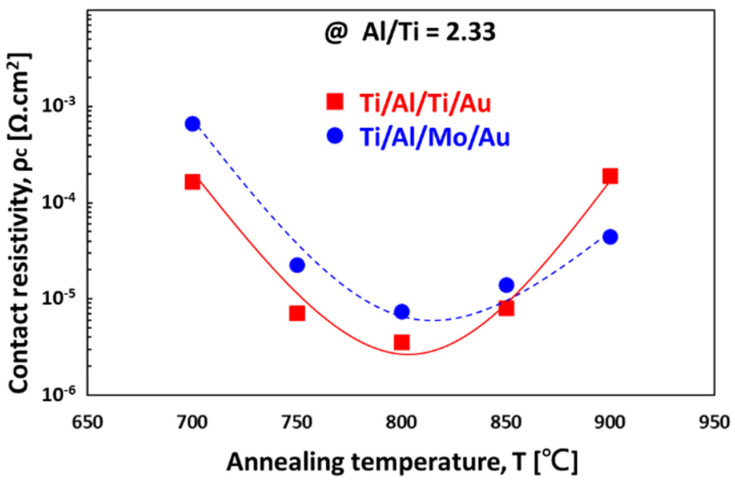
Ti/Al-based ohmic contacts on AlGaN/GaN HEMT as a function of annealing temperature [66]. (Data from [66]).

As shown in Figure 10, the contact resistivity of GaN can reach 5 × 10^−6^ Ω cm^2^ after annealing a Ti/Al-based (Ti/Al = 0.43) ohmic contact metal at 800 °C [66]. The effect of the ratio of Ti and Al thicknesses is also discussed in the literature [67]. By summarizing the studies from many research works, it can be shown that Ti/Al ohmic contact metals are, thus far, the most stable ohmic contacts for n-type GaN, and the major factors that affect the contact resistances are the Ti/Al ratio and the annealing conditions. As long as the top conductive Au metal is well separated from the Ti/Al layer by a diffusion barrier, the contact resistivity is not affected by the Au conductive layer [66,67].

### 2.3. Schottky Metal Gate

**Figure 11:**
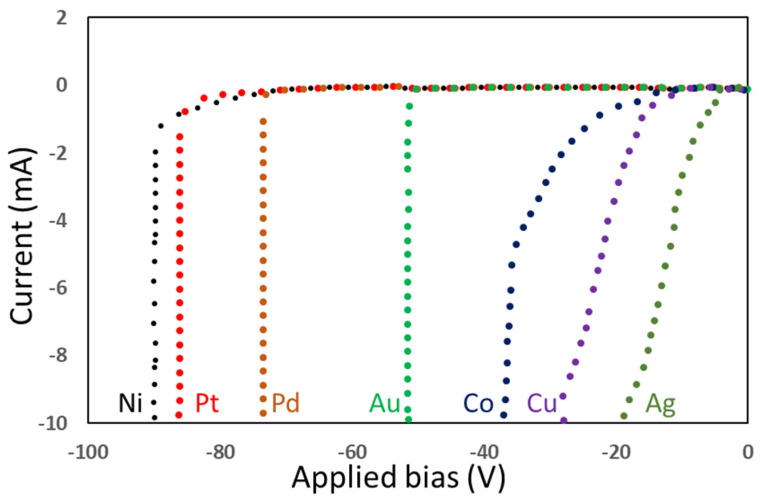
IV characteristic diagram of different metals under reverse bias [68]. (Data from [68]).

The choice of gate metal also has significant impacts on the performance and reliability of GaN HEMTs. In the structure of a typical GaN HEMT, there is a wide-bandgap AlGaN barrier layer between the gate metal and the GaN channel. The gate metal forms a Schottky contact on the AlGaN barrier layer which can control the polarization charge density at the AlGaN/GaN interface. The charge density of the heterostructure and the drain current is controlled by modulating the Schottky contact with the applied gate voltage. An excessive gate leakage current is not allowed for HEMT devices because it could result in undesired power consumption at the gate or incomplete channel closure. As most GaN HEMTs are “normally on” devices owing to the intrinsic characteristic of the AlGaN/GaN heterostructure, the drain current of GaN HEMTs has to be turned off by setting the Schottky gate diode at a reversed bias. As shown in Figure 11, Pd, Ni, and Pt are reported to be suitable gate metals since the leakage currents of these metals are the smallest when the GaN Schottky gate diodes are negatively biased [68].

On the other hand, the channel temperature can be high during the on-state operation of a power device. Therefore, high thermal stability is required for the selected gate metal. Ni has shown excellent stability [69,70]; thus, Ni/Au is currently one of the most commonly used gate metals. WN is another gate metal showing good stability and low leakage. Researchers found that a WN gate can be formed by annealing a W gate in a N_2_ atmosphere [71]. In addition, metal–insulator–semiconductor (MIS) gates are adopted to more effectively reduce gate leakage. The MIS-HEMT structure will be discussed in more detail in Section 3.2.

## 3. CMOS-Compatible Au-Free GaN Technology

In this section, we will discuss the new developments in structures and materials for CMOS-compatible process technology in the ohmic contact process and gate process.

**Figure 12:**
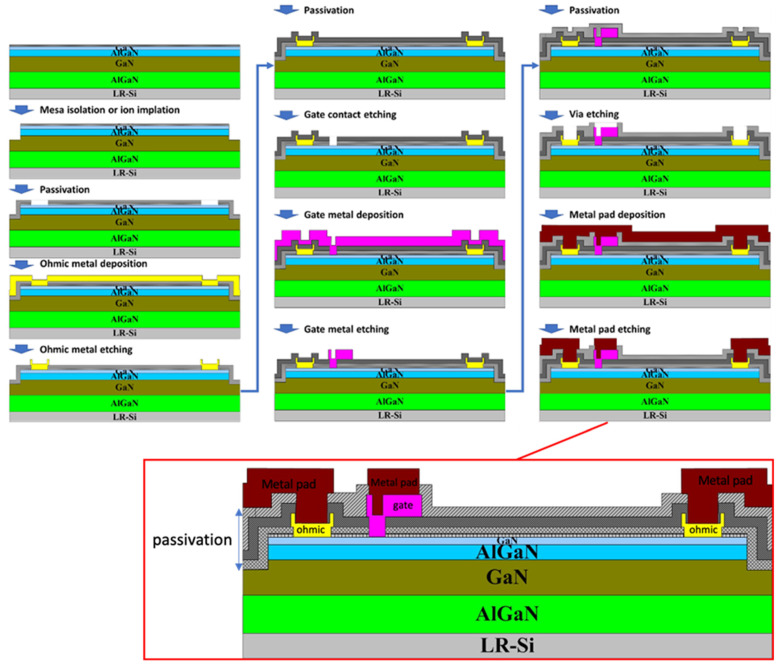
Au-free CMOS-compatible process.

Traditional III–V power semiconductor devices use Au in the ohmic metal, gate metal, and interconnect. The evaporation/lift-off process is used to fabricate the metallization structure, but it is not compatible with the CMOS production line. Metallization of CMOS chips usually adopts interlevel dielectrics to define and isolate metal lines. Metal is then filled into the desired trench areas, and the metal deposited at undesired areas is removed by etching or CMP processes. The typical process flow of a CMOS-compatible process is depicted in Figure 12. The commonly used metals are Ti, TiN, Al, Cu, W, or TaN. Moreover, most manufacturers are in the process of scaling up the GaN production wafer size to 200 mm or 300 mm. Therefore, major companies (for example, Intel, IMEC) have invested in the development of a gold-free process [72,73,74].

### 3.1. Ohmic Contact

**Figure 13:**
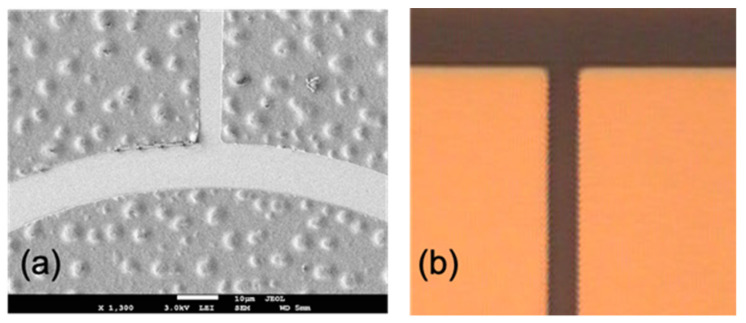
SEM image of (**a**) Au-based Ti/Al/Ni/Au ohmic contact annealed at 900 °C [75], and (**b**) Au-free ohmic contact (Ti/Al/Ti/TiN) annealed at 950 °C. Figure reproduced with permission from AIP Publishing.

Traditional III–V devices mostly use Au-based metallization schemes that are incompatible with CMOS. The commonly used Ti/Al/Ni/Au ohmic contact metal for GaN can be replaced by an MOS-compatible metal stack such as Ti/Al/Ti/TiN. A Au-based Ti/Al/Ni/Au ohmic contact metal after high-temperature rapid thermal annealing (800~900 °C) usually shows a rough surface roughness, as shown in Figure 13a. After high-temperature annealing, the upper Au layer and Al will react and form a rough surface [75]. The rough ohmic metal surface may affect lithography alignment and subsequent integration. As shown in Figure 13b, the Au-free Ti/Al/Ti/TiN ohmic metal still shows a smooth surface and exhibits low contact resistivity of 1 × 10^−5^ Ω cm^2^ after 950 °C high-temperature annealing.

**Figure 14:**
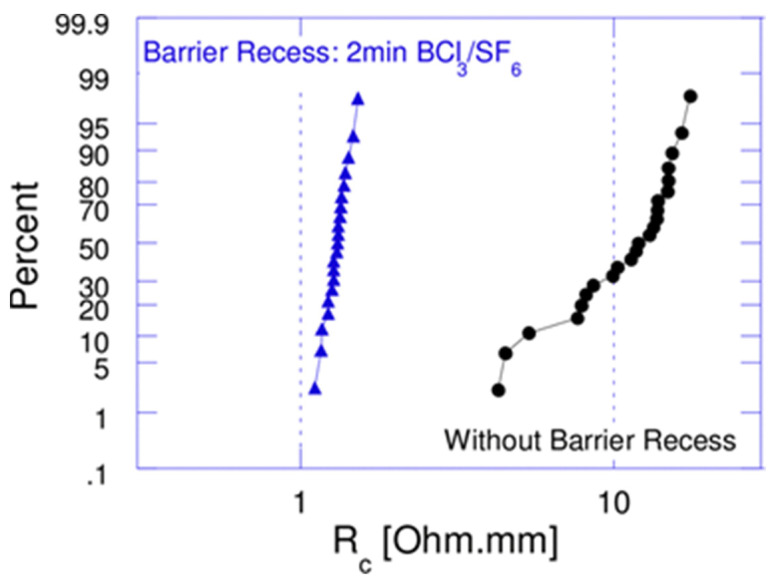
R_C_ distributions of 200 mm GaN HEMT wafers with and without AlGaN barrier recess annealed at 550 °C [72]. Figure reproduced with permission from IEEE 2012 24th International Symposium on Power Semiconductor Devices and ICs.

In addition, the formation of ohmic contacts to GaN usually relies on metal diffusion to form a nitrogen vacancy-rich heavily n-doped interface, as described in Section 2.2. However, the diffusion of an ohmic metal through the AlGaN barrier layer requires annealing at a relatively high temperature (>800 °C). One method to reduce the alloying temperature is to perform the AlGaN recess etch at the ohmic contact area before the Ti/Al ohmic metal is deposited. Figure 14 shows the R_c_ reduction of a device with an AlGaN recess etched at the ohmic region [72], and the Ti/Al/Ti/TiN ohmic contact metal alloyed at 550 °C exhibits excellent contact resistivity.

**Figure 15:**
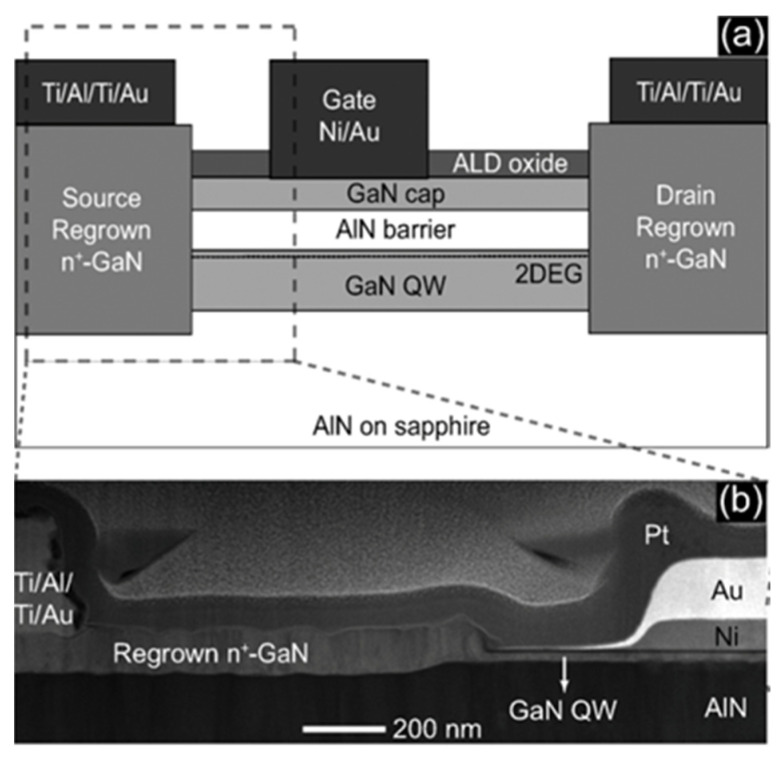
(**a**) Device structures of GaN HEMT with regrown ohmic layer. (**b**) STEM image showing regrown GaN connected to the GaN QW channel [85]. Figure reproduced with permission from IEEE Electron Device Lett.

In addition to the surface morphology, the line edge is usually not very smooth after the high-temperature diffusion process. Moreover, it is desired to further reduce the contact resistivity of the ohmic contact to GaN. Regrowth of n+ GaN at the ohmic contact area is a feasible method to further reduce the contact resistance and enable non-alloy ohmic contact to obtain a better surface morphology and interface [76,77,78,79]. After AlGaN recess etching is performed at the ohmic contact region, an n-type heavily doped GaN layer is epitaxially grown at the ohmic contact region by MOCVD. Then, Ti/Al or TiN is deposited as a non-alloy ohmic contact metal [41,80,81,82,83]. Several research groups have reported promising results, and a contact resistivity as low as 1.6 × 10^−5^ Ω cm^2^ can be achieved [84]. Figure 15 [85] is a STEM micrograph showing the good epitaxial quality of a regrown ohmic layer.

**Figure 16:**
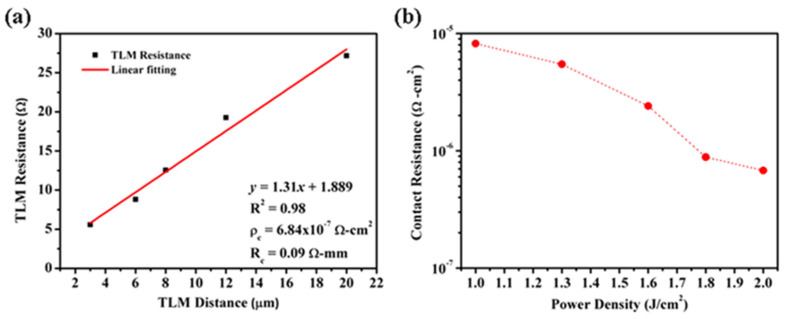
(**a**) The contact resistance of implanted sample pulsed laser annealing shows a low contact resistance of 6.84 × 10^−7^ Ω cm^2^. (**b**) The contact resistance under different power densities of pulsed laser annealing [86]. (Data from [86]).

Using ion implantation and laser annealing technology to form non-alloy ohmic contacts on GaN was reported recently. The power density of the pulsed laser annealing is optimized to activate the Si ion-implanted GaN, and then the Ti/Al/Ni/Au ohmic is annealed at 500 °C to form an ohmic contact. Experimental results show that a smooth surface can be obtained and results in a comparable contact resistance; the contact resistivity of a wafer processed using ion implantation/laser annealing technology is shown in Figure 16 [86].

### 3.2. Metal–Insulator–Semiconductor (MIS) Gate

**Figure 17:**
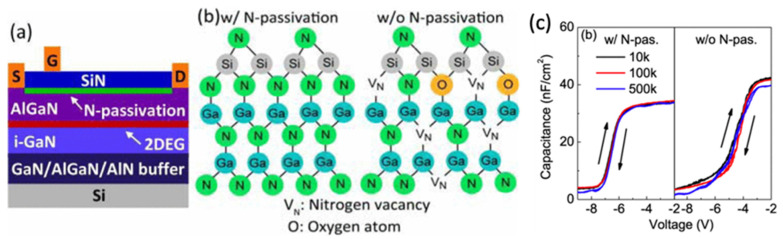
(**a**) Cross-section of the AlGaN/GaN HEMT with N passivation and 20 nm SiN gate dielectric layer. (**b**) Schematic of the atomic arrangement at SiN/GaN interface with and without N passivation. (**c**) C–V characteristics of SiN/GaN MIS capacitor with and without N passivation at different frequencies varying from 10 to 500 kHz [87]. Figure reproduced with permission from IEEE Electron Device Lett.

The traditional gate process of GaN HEMTs uses a Schottky metal to modulate the 2DEG in the channel. As mentioned earlier, Ni/Au is the most commonly used Schottky gate metal. For Au-free CMOS-compatible processes, a CMOS-compatible high-k gate dielectric, such as CVD-deposited SiN, atomic layer deposition (ALD)-deposited aluminum oxide (Al_2_O_3_), or hafnium oxide (HfO_2_), is placed between the AlGaN barrier layer and the gate metal to form an MIS-HEMT structure to better control the gate leakage current [87,88]. With proper surface treatment, the metal–insulator–semiconductor (MIS) gate shows good stability, and it can provide additional protection to the device surface. Figure 17 shows the device structure of a SiN gate MIS-HEMT and the low hysteresis C–V behavior of the gate. It was also reported that low-temperature deposited SiN_X_ combined with high-temperature deposited LPCVD SiN can form a robust MIS gate for GaN power HEMT application [89].

**Figure 18:**
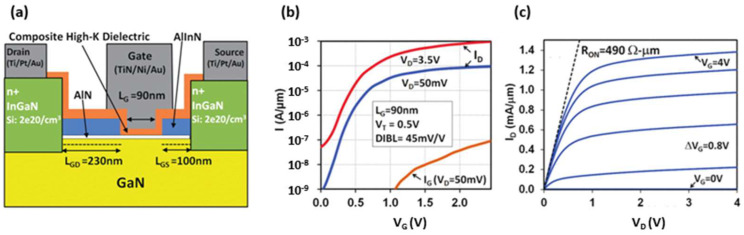
(**a**) Schematic of the e-mode high-K GaN MOS-HEMT. (**b**) I_D_-V_G_ of the L_G_ = 90 nm e-mode GaN MOS-HEMT showing low I_OFF_ = 70 nA/μm (at V_G_ = 0 V, V_D_ = 3.5 V), and (**c**) I_D_-V_D_ of the same device showing low on-resistance of R_ON_ = 490 Ω μm [24]. Figure reproduced with permission from IEEE 2015 Symposium on VLSI Technology.

Recently, Intel reported the fabrication of an enhancement-mode (e-mode) GaN MOS-HEMT with an AlN/Al_2_O_3_/HfO_2_ composite high-K gate dielectric which shows an outstanding f_T_/f_MAX_ of 200 GHz/300 GHz, as mentioned in Section 1.1. These results show that the GaN MOS-HEMT is attractive for realizing energy-efficient, compact voltage regulators and RF power amplifiers for mobile systems on a chip (SoCs) [24,33], as shown in Figure 18.

## 4. Recent Trend in Power GaN

Applications in power electronics represent the largest market for GaN electronic devices currently. Major trends in the developments for GaN power devices are p-GaN HEMTs for enhancement-mode (E-mode) operation and GaN-on-GaN technology for higher operating voltages. The breakdown voltages for p-GaN HEMTs and vertical GaN-on-GaN devices have exceeded 1000 V (@R_on,sp_ of 2 mΩ cm^2^) and 1.5 kV (@R_on,sp_ of 1 mΩ cm^2^), respectively. We will describe these trends in the following paragraphs.

### 4.1. p-GaN Technology

As stated in Section 1, the presence of a 2DEG in a high-electron mobility channel makes the GaN HEMT a normally on device. In the case of power switching applications, a normally on (depletion mode) GaN HEMT is less desirable than a normally off (enhancement-mode) GaN HEMT due to the fail-safe operation of the former and simpler gate control of the latter. A single-chip e-mode GaN HEMT can be fabricated using p-GaN gate [90,91,92,93,94], gate recess [94,95,96], or plasma treatment techniques [97,98]. In terms of performance, manufacturability, and reliability, the p-GaN gate HEMT showed a good balance which has resulted in the first commercialization of single-chip e-mode GaN devices [99,100,101]. 

**Figure 19:**
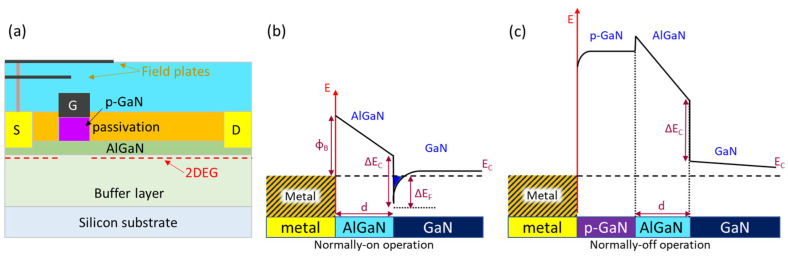
(**a**) Cross-sectional schematic of p-GaN gate HEMT [102] and (**b**) schematic of the operation principle of the normally on HEMT and (**c**) normally off HEMT [99]. (Data from [99,102]).

The cross-sectional device structure of the p-GaN gate HEMT is shown in Figure 19a [102]. The band structures of normally on AlGaN/GaN HEMTs and normally off p-GaN/AlGaN/GaN HEMTs are shown in Figure 19b,c [99]. The high-electron mobility 2DEG channel is depleted at a zero-bias condition in the case of the p-GaN gate HEMT as the conduction band energy of AlGaN is lifted due to the presence of the p-GaN layer. The device characteristics of the p-GaN gate HEMT including V_TH_, the V_GS_ limitation, and the gate leakage current (I_GSS_) depend on the structure of the gate stack which could vary according to different manufacturers [98,99,100,101,102]. Table 2 shows the values of gate characteristics of different p-GaN devices that have emerged recently in the market [102]. 

To effectively deplete the 2DEG channel at V_G_ = 0, the typical AlGaN thickness is 10~15 nm, and the thickness of the p-GaN gate is around 50~100 nm. The typical p-type dopant for the p-GaN (or p-AlGaN) gate is Mg with a doping concentration of 10^18^~10^19^ cm^−3^. Two major process issues are critical for the p-GaN gate HEMT device. The first is the selective etch to remove p-GaN from areas other than gates. Plasma damage at the recessed GaN surface can cause a problem of reliability. The other issue is the higher gate leakage. Since the p-GaN gate is under a high electric field at both positive and negative gate voltages, when the gate voltage is positive, it is depleted and stressed from the gate metal side. When the gate voltage is negative, it is depleted and stressed from the channel side. Therefore, the p-GaN epitaxy quality and p-GaN/metal interface have to be very robust to avoid a larger gate leakage under high-voltage stress [99].

### 4.2. GaN-on-GaN Technology

At present, the lateral heterojunction AlGaN/GaN HEMT grown on the Si substrate dominates the development of commercial GaN power electronic devices (up to 10 kW) [89]. Lateral devices have shown great potential and are becoming mature [95,103,104,105], whereas vertical topologies are still in their infancy [106]. However, lateral devices become unappealing in both cost and manufacturability when the power is very high since it requires a large chip area. To manufacture such high-power devices, vertical topologies are preferred as the chip areas of the devices are smaller than lateral devices owing to vertical devices’ ability to withstand the high blocking voltage in the vertical direction into the bulk material.

**Figure 20:**
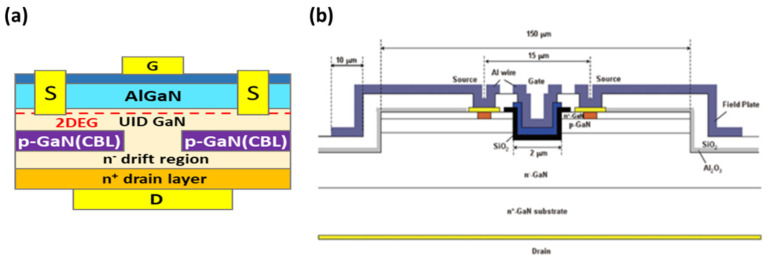
Cross-sectional structure of (**a**) CAVET [107] and (**b**) vertical GaN trench MOSFET [108]. Figure reproduced with permission from Japan Society of Applied Physics.

Unlike lateral devices being grown on SiC or Si substrates, vertical devices need to be grown on GaN bulk substrates. One typical vertical GaN-on-GaN HEMT device is the current aperture vertical electron transistor (CAVET) [107] shown in Figure 20a. The 2D electron gas channel formed at the interface between AlGaN and GaN is used in conjunction with the bulk GaN drift region to achieve low on-resistance. Since the blocking voltage of the device is sustained in a vertical direction into the bulk of the device, the breakdown voltage at the same specific current can be much higher than the HEMT. In this type of device, planar gates control the electron flow in the 2DEG channel, and electrons will then flow vertically towards the n-GaN drift region through a conductive aperture between the current blocking layers (CBLs), thereby holding the blocking voltage into the device bulk. Another type of vertical GaN device is the vertical GaN trench MOSFET [108]. As depicted in Figure 20b, the gate trench is buried into the p-type channel layer. The inversion channel is formed at the sidewall of the trench gate during the “on” state. Then, the thick bulk n-drift region conducts the current to the bottom drain electrode. As mentioned previously, such types of devices have been shown to sustain a voltage above 1.5 kV. The requirement of extensive passivation and field planting in a lateral device such as an HEMT was eliminated in the case of the vertical device due to the presence of a high electric field inside the bulk which also alleviates current collapse due to surface traps.

## 5. Conclusions

In the past decade, the wide application of GaN electronic devices has attracted a lot of attention and has gradually matured. Their applications can range from radio frequency power amplifiers to power electronic systems. Due to their wide range of applications and acceptance in the market, the manufacturing process has also evolved from the traditional Au-based III–V technology to high-volume and high-yield CMOS-compatible technology. Moreover, the development of high-voltage vertical device structures has become a new direction. In this article, we provide a short and comprehensive overview to introduce these important technological developments. Due to important applications for such fifth-generation mobile communications, wireless high-speed chargers, and electric vehicles, we expect the technology and output value of GaN electronic devices to flourish and grow rapidly in the foreseeable future.

## Figures and Tables

**Table 1:** The lattice and thermal mismatch of Si, SiC, Sapphire, AlN, and GaN [46].

Mismatch	Si	SiC	Sapphire	AlN	GaN
Crystal Structure	FCC	HCP	HCP	HCP	HCP
Lattice Constant (Å)	5.43	3.08	4.758	3.112	3.189
Lattice Mismatch (%)	−16.9	3.5	16.08	2.4	-
Thermal Expansion (10^−6^ K)	3.59	4.3	7.3	4.15	5.59
Thermal Mismatch (%)	55	30	−23	34	-

**Table 2:** Gate characteristic of different p-GaN devices [102].

Manufacturer	V_TH_ (V)	I_D_/I_G_ at V_GS_ = 6 V	VGS_min_(V)/ VGS_max_(V)	Gate Drive Voltage(V)
EPC	1.4	10^3^–10^4^	−4/6	4–5
Panasonic	1.2	10^2^–10^3^ *	−10/4.5	3–5
GaN Systems	1.3	10^5^–10^6^	−10/7	5–6.5
IMEC	~2.0	10^5^–10^10^	−/<12	-
FBH Berlin	~1.0	10^3^–10^4^	−/~7	5

* Data obtained under V_GS_ = 4.5 V, the maximum allowable gate voltage for the Panasonic device.

## Data Availability

Not applicable.
